# Numerical prediction of thrombus risk in an anatomically dilated left ventricle: the effect of inflow cannula designs

**DOI:** 10.1186/s12938-016-0262-2

**Published:** 2016-12-28

**Authors:** Sam Liao, Benjamin Simpson, Michael Neidlin, Tim A. S. Kaufmann, Zhiyong Li, Maria A. Woodruff, Shaun D. Gregory

**Affiliations:** 1 Innovative Cardiovascular Engineering and Technology Laboratory (ICETLAB), Critical Care Research Group, The Prince Charles Hospital, Chermside, QLD 4032 Australia; 2 Institute of Health and Biomedical Innovation (IHBI), Queensland University of Technology (QUT), Kelvin Grove, QLD 4059 Australia; 3 School of Engineering, Griffith University, Southport, QLD 4215 Australia; 4 Department of Cardiovascular Engineering, Institute of Applied Medical Engineering, Helmholtz Institute, RWTH Aachen University, Aachen, Germany

**Keywords:** Heart failure, Left ventricular assist device, Multiphase modelling, Patient-specific

## Abstract

**Background:**

Implantation of a rotary blood pump (RBP) can cause non-physiological flow fields in the left ventricle (LV) which may trigger thrombosis. Different inflow cannula geometry can affect LV flow fields. The aim of this study was to determine the effect of inflow cannula geometry on intraventricular flow under full LV support in a patient specific model.

**Methods:**

Computed tomography angiography imaging of the LV was performed on a RBP candidate to develop a patient-specific model. Five inflow cannulae were evaluated, which were modelled on those used clinically or under development. The inflow cannulae are described as a crown like tip, thin walled tubular tip, large filleted tip, trumpet like tip and an inferiorly flared cannula. Placement of the inflow cannula was at the LV apex with the central axis intersecting the centre of the mitral valve. Full support was simulated by prescribing 5 l/min across the mitral valve. Thrombus risk was evaluated by identifying regions of stagnation. Rate of LV washout was assessed using a volume of fluid model. Relative haemolysis index and blood residence time was calculated using an Eulerian approach.

**Results:**

The inferiorly flared inflow cannula had the lowest thrombus risk due to low stagnation volumes. All cannulae had similar rates of LV washout and blood residence time. The crown like tip and thin walled tubular tip resulted in relatively higher blood damage indices within the LV.

**Conclusion:**

Changes in intraventricular flow due to variances in cannula geometry resulted in different stagnation volumes. Cannula geometry does not appreciably affect LV washout rates and blood residence time. The patient specific, full support computational fluid dynamic model provided a repeatable platform to investigate the effects of inflow cannula geometry on intraventricular flow.

## Background

The number of patients receiving left ventricular assist devices (LVADs) to treat heart failure is on the rise, with over 2500 devices implanted per year in the US alone [1]. Inflow attachment of LVADs is generally achieved via the left ventricular apex with a special tube called an inflow cannula (IC), while outflow attachment is generally achieved with a graft attached to the ascending aorta. Insertion of a foreign object into the left ventricle (LV) can affect intraventricular flow. This disruption to ventricular haemodynamics can potentially cause thrombus formation due to stagnation zones, recirculation zones and high shear stresses [2–7]. Currently, no gold standard exists for the IC geometry, with each clinically used device having a custom design.

To minimise thrombus formation, it is beneficial to have low LV blood residence time (BRT), low stagnation and efficient LV washout. Blood stagnation and recirculation can be related to low velocities and shear rates [2]. Studies have shown that the native haemodynamics inside the LV are asymmetric, unsteady, and have a large diastolic vortex that directs blood towards the aortic valve [8–11]. However, most computational fluid dynamic (CFD) studies of the LV have been extensively simplified [12–16]. Most models do not consider recreating natural ventricular geometry, haemodynamics and the corresponding effects of an IC. This could significantly alter the areas where thrombus formation may occur.

Inflow cannula geometry, insertion depth, insertion angle and position may all alter the intraventricular flow fields. One study has shown that by increasing the IC insertion length from 24 to 34 mm, a higher survival rate of 63.5% compared to 52.9% was observed [17]. This minor difference in insertion length can greatly affect the patient´s outcome; however, the exact reason is unknown. The incidence of stroke was found to be 23.2 and 3.8% with the short and long cannula, respectively [17]. This significant drop in neurological adverse events could stem from a range of contributing factors including patient specific causes, surgical technique and blood flow patterns. It was hypothesised that the difference in neurological events could be linked to poor LV washout and stagnation regions caused by the shorter IC.

While there are several studies reporting on intraventricular flow dynamics with LVAD support, these are limited by the lack of anatomically correct ventricular geometry. For instance, Loerakker et al. [12] and Ong et al. [13] simulated the LV geometry using a prolate ellipsoid with tubes either side to represent the atrium, aorta and LVAD IC. Ong et al. [13] studied the effect of cannula placement on thrombosis. Three different insertion lengths of a trumpet tipped inflow cannula were simulated where the tip was near the apex, inserted one-fourth of the LV length, or inserted half of the LV length. The risk of thrombosis was evaluated by intraventricular vorticity distributions, intensities, stagnations and wall shear stresses. It was found that the cannula inserted one-fourth into the LV resulted in negligible fluid stagnation as it created higher vortex intensities. Even though a deformable wall was implemented, the non-physiological intraventricular flows provided by the inlet boundary condition and ventricular geometry could affect the results significantly.

Liu et al. [14] used a conical volume with no valves to represent the LV to investigate 4 different types of cannulae: blunt, bevelled, trumpet and caged. Blood compatibility was evaluated based on wall shear stress and exposure time, of which none met the estimated criteria for haemolysis. It was found that the trumpet design had the best blood compatibility, comparatively.

An analysis of thrombosis potential in LVAD drainage cannulae was investigated by Fraser et al. [18]. The study implemented three clinically available, at the time of writing, cannulae (Medtronic DLP 12, 16 and 24 F) in an MRI segmented LV model. Different flow rates were used, applied at a constant uniform velocity. It was found that 12 and 16 F cannulae were superior due to lower fluid stagnation volumes compared to the 24 F cannula at flows below 0.75 l/min.

The impact of mitral valve (MV) modelling should also not be underestimated, as this dictates the inlet flow to the ventricle. Others have either ignored the MV entirely [12–14] or modelled it using rigid plates [15]. Most limitations in previous studies arose from numerical models that could not recreate physiological LV haemodynamics, generally attributed to over-simplification. Therefore, a model which combines anatomically correct ventricular and mitral valve geometry would provide beneficial evaluation of LVAD IC geometry. The aim of this study was to create a patient-specific LV with an approximated MV model to determine the effects of various IC geometries on intraventricular flow in a total heart failure simulation. It was hypothesised that a cannula with a smoother transition from the cannula to the endocardium can reduce regions of stagnant flow and increase LV washout.

## Methods

### Patient data

**Fig. 1:**
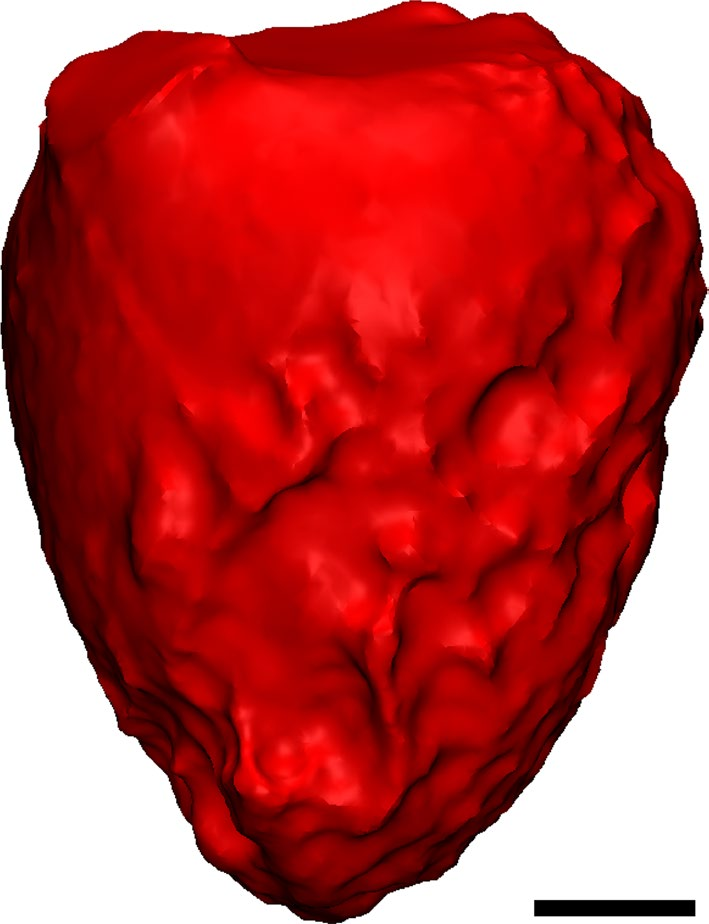
Extracted and processed model of a dilated patient specific left ventricle from CT imaging data. *Scale bar* represents 20 mm

Computed tomography (CT) angiography was performed on a de-identified rotary blood pump candidate. CT imaging data (335 slices with a thickness of 0.75 mm per slice) was used to extract the LV volume using Mimics (Materialise, Belgium NV). The extracted LV volume was the end systolic volume. In this study, a total heart failure model was used, indicating zero ventricular contractility and, therefore, zero wall motion. Smoothing was completed in 3-matic with all internal voids filled, as seen in Fig. 1. The model was exported as .iges files for manipulation in Solidworks 2015 (Dassault Systèmes SE, Vélizy-Villacoublay, France).

The left atrium was represented by a 40 mm diameter cylinder with the MV placed adjacent to the aortic valve with guidance from CT data. The aortic valve was not included in the model due to full support by the LVAD: the aortic valve is always closed.

### Mitral valve modelling

**Fig. 2:**
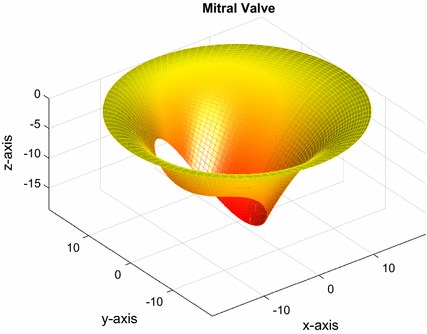
Parametric approximation of a mitral valve opened at 45°. Axes are in units of mm. *Coloured shading* has been included for visualisation purposes

The MV surface was approximated using a set of parametric equations, as described by Domenichini et al. [19]. The equations are as follows:

$$x_{v} \left( {\theta ,s} \right) = R\cos \theta \left( {1 - s\cos \varphi } \right) - \varepsilon Rs\cos \varphi$$

$$y_{v} \left( {\theta ,s} \right) = R\sin \theta \,\left( {1 - sk\cos \varphi } \right)$$

$$z_{v} \left( {\theta ,s} \right) = - s^{2} \left( {\frac{1 + k}{2} + \varepsilon \cos \theta + \frac{1 - k}{2}\cos 2\theta } \right)R\sin \varphi$$

where θ was 100 linearly separated points from 0 to 2π; s was 40 linearly separated points from 0 to 1; *ε* = 0.35 described the symmetry ratio between the anterior and posterior leaflets; *k* = 0.6 described the ellipticity of the valvular edge; $\varphi = \frac{\pi }{4}$ rads described the MV opening angle; and R = 19.5 mm defined the radius of the MV. The radius of the MV was determined by evaluating the patient specific model and fitting the most appropriately sized valve. Generation of the surface plot was performed in MATLAB R2015a (MathWorks, Natick, Massachusetts, United States), as shown in Fig. 2. The MV surface was converted to a .stl file, in MATLAB R2015a, for manipulation in Solidworks 2015 (Dassault Systèmes SE, Vélizy-Villacoublay, France). As the parametric surface does not generate an infinite number of segments for a true circle, which was needed to mate with the cylinder (left atrium), the approximated MV surface diameter was increased from 39 to 40 mm in Solidworks 2015.

### Cannula geometries

**Fig. 3:**
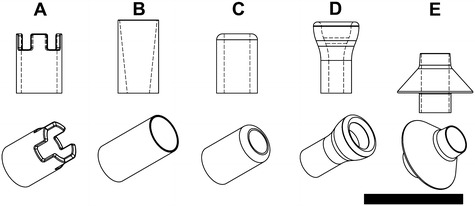
Five different cannula geometries are shown with a side and an isometric view. Reference identifiers for each cannula geometry is as labelled. *Scale bar* represents 50 mm

Five different cannula geometries were modelled based on both clinically available designs and a suture-less design which is currently under development. The working drawings of these cannulae can be seen in Fig. 3. Each cannula has been assigned an identification letter for the ease of referencing in this paper. A brief description of the cannulae are as follows: (A) a crown tipped cannula (B) a relatively long and sharp tipped cannula (C) a short tubular cannula with a large inlet fillet, (D) a trumpet like tip, and (E) an inferiorly flared cannula. Alignment was performed by intersecting the central longitudinal axis of the cannula with the origin of the MV. Based on clinically available cannulae of similar geometry, cannula lengths were 30, 37, 30, 36 and 20 mm for cannulae A, B, C, D and E, respectively, which was inserted completely inside the LV.

### Meshing

**Fig. 4:**
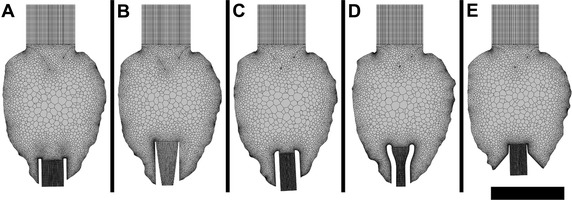
Meshing of cannulated LV models **A**, **B**, **C**, **D** and **E**. *Inlets* and *outlets* consisted of hexahedral cells with polyhedral cells for the LV. *Scale bar* represents 60 mm

Mesh generation was completed using ANSYS Meshing (ANSYS 16.1, Inc., Cecil Township, Pennsylvania, U.S.) and Fluent (ANSYS 16.1, Inc., Cecil Township, Pennsylvania, U.S.). Using recorded selective meshing, a multizone method was used to create hexahedral cells at the inlet and outlet first, then the LV was meshed with patch conforming tetrahedral cells. The mesh was imported into Fluent and the tetrahedral cells were converted to polyhedral cells, which improved convergence and solution times. Cross section of the mesh structure for each model can be found in Fig. 4, showing the hexahedral cells at the inlet and outlets with polyhedral cells within the LV. The number of cells and cell quality can be seen in Table 1. The variation in cell numbers between the models were predominantly attributed to the inflow cannula geometry. For example, higher mesh counts were required for cannula A due to the greater geometrical complexity. A mesh independence study was conducted with cannula C. Three different cell counts were used: coarse (787,682), medium (1,575,093) and fine (1,921,958). A transverse plane placed 60 mm above the apex was recorded for area-weighted average velocity. It was found that there was a change of less than 5% between the medium and fine model in terms of the median velocity. As a result, it was deemed that all cell counts above the fine model was appropriate.

**Table 1:** Mesh statistics, as reported by ANSYS fluent

Model	Number of cells	Minimum orthogonal quality	Maximum skewness
A	2,670,464	0.205	0.795
B	1,921,958	0.221	0.779
C	2,343,856	0.123	0.877
D	2,071,986	0.228	0.772
E	2,115,856	0.207	0.793

### Boundary conditions

Blood was defined to be Newtonian fluid with a viscosity and density of 0.0035 Pa s and 1059 kg/m^3^, respectively. A constant flow rate of 5 l/min was prescribed at the inlet (atrium). Constant flow was chosen to represent full LVAD support with no native ejection through the aortic valve. It was assumed that whichever rotary LVAD is selected, the rotational speed would be set to achieve a flow rate of 5 l/min. This flow rate was equivalent to a bulk mass flow rate of 0.088 kg/s with the flow prescribed normal to the boundary. The cannula outlet had a 0 Pa pressure outlet. All walls were defined with a no slip condition.

### Solution algorithm

A finite volume method was used to solve the mass and momentum conservation equations of the incompressible Newtonian fluid. The Reynolds number was calculated at the inlet as follow:

$$Re = \frac{\rho uD}{\mu }$$

where *ρ* [m^3^/kg] is the density, *u* [m/s] is the velocity, *D* [m] is the hydraulic diameter and *µ* [Nm/s] the dynamic viscosity.

$$Re = \frac{1059 \times 0.066 \times 0.04}{0.0035}$$

$$Re \approx 800$$

From the low *Re* number, a laminar model was used. ANSYS Fluent was used for the calculations. The Pressure Implicit with Splitting of Operator (PISO) pressure–velocity coupling scheme was selected. Spatial discretisation for pressure, momentum and volume fraction was defined to be a second order upwind scheme. A transient simulation of 20 s was performed. Time integration was done using a bounded second order implicit backward Euler method with time steps of 0.002 s. Results for analysis were taken from the last 15 s. The first 5 s allowed the flow field to be established. Results were deemed to be converged when the scaled residuals were below 10^−4^ for continuity, x velocity, y velocity and z velocity. Each time step required approximately 15–20 iterations. Using 16 CPUs per model, each simulation took approximately 1.5 weeks to complete. All calculations were performed on a High Performance Computing cluster (Queensland University of Technology, Brisbane, Australia).

### Stagnation regions

There is currently no standard in the definition of stagnation volume [13, 14, 20, 21]. Regions of potential thrombus formation were defined by volumes of low velocity magnitudes, less than 0.001 m/s, and strain rates of less than 2 s^−1^ [18]. The additional low strain rate criteria prevented the inclusion of low velocity magnitudes experienced at the walls. This criteria was used to relatively compare the models. All cell volumes that met the criteria were assigned a value of 1, all others were assigned 0. A plane was defined 50 mm above the apex and all volumes below this plane were defined as a volume of interest. This was used for calculation of thrombus risk (TR) volumes around the cannula. The TR volume was calculated by integrating the assigned cell value, 0 or 1, over the volume of interest. Evaluation of the stagnation regions was performed at time points 0, 5, 10 and 15 s. At each time point, an image was captured that resulted in four images per cannula. Using imaging processing, the median channel values for each pixel of the four images were taken, resulting in removal of stagnation regions that were intermittent. This allowed clearer qualitative comparisons between the cannulae.

#### Volume of fluid modelling

Volume of fluid (VOF) modelling, as developed by Sonntag et al. [22], was used to determine LV washout. The VOF model is generally used for interface tracking of immiscible fluids. However, in this study the VOF model was used to calculate displacement of one fluid (old blood) by another fluid (new blood), and so the primary and secondary phase had identical fluid properties. An implicit formulation was applied with dispersed interface modelling. The secondary phase was started after 5 s of flow stabilisation. The pressure outlet had a secondary phase backflow volume fraction of 0, which prevents “new blood” from entering from the outlet. The time taken for LV washout was calculated by taking the volume integral of the volume fraction of each cell, which ranged between 0 and 1. If the initial phase (old blood) was of interest, then a volume fraction of 1 indicates the cell is full of old blood and conversely, 0 indicates a cell has no old blood. A value of 0.5 indicates the surface between the new and old blood. The rate of LV washout was determined by integrating the volume fraction, between 0 and 1, over the cell volume. This was normalised with the total volume of the entire domain.

### Haemolysis index

An Eulerian approach was implemented to estimate the blood damage potential due to different inflow cannula geometries. A scalar variable, hb’ equal to hb^1/α^, represented the plasma free haemoglobin as a percentage of the total blood haemoglobin [23, 24]. The scalar transport equation is as follow:

$$\frac{{d\left( {hb^{'} } \right)}}{dt} + v\rho \nabla \left( {\Delta hb^{'} } \right) = S$$

where S is the source term defined as:

$$S = \rho \left( {C\tau^{\beta } } \right)^{1/\alpha }$$

The constants used in the source term have been found by Giersiepn et al. [25], where *C* = 3.62 × 10^−5^, *β* = 2.416 and *α* = 0.785. Shear stress, *τ*, was calculated by multiplying the strain rate at each cell by the laminar viscosity. The source term was applied using a user defined scalar. Simplified, the haemolysis index, HI, calculation can be expressed as:

$$HI_{n}^{{\frac{1}{0.785}}} = HI_{n - 1}^{{\frac{1}{0.785}}} + \left( {3.62 \times 10^{ - 5} \times \tau^{2.416} } \right)^{{\frac{1}{0.785}}} dt$$

where n is the current state and n−1 is the previous state. To assess the effect of inflow cannula geometry on the LV flow and consequent HI, a transverse plane 60 mm above the apex was implemented with the mass-weighted HI calculated with the HI evaluated at the last time point. The HI was not calculated at the outlet as the main goal was to assess the effect of cannula geometry on intraventricular flow. Furthermore, a similar Eulerian approach was used to determine blood residence time. The area-weighted average blood residence time was calculated at the cannula outlet.

## Results

### Velocity distribution

**Fig. 5:**
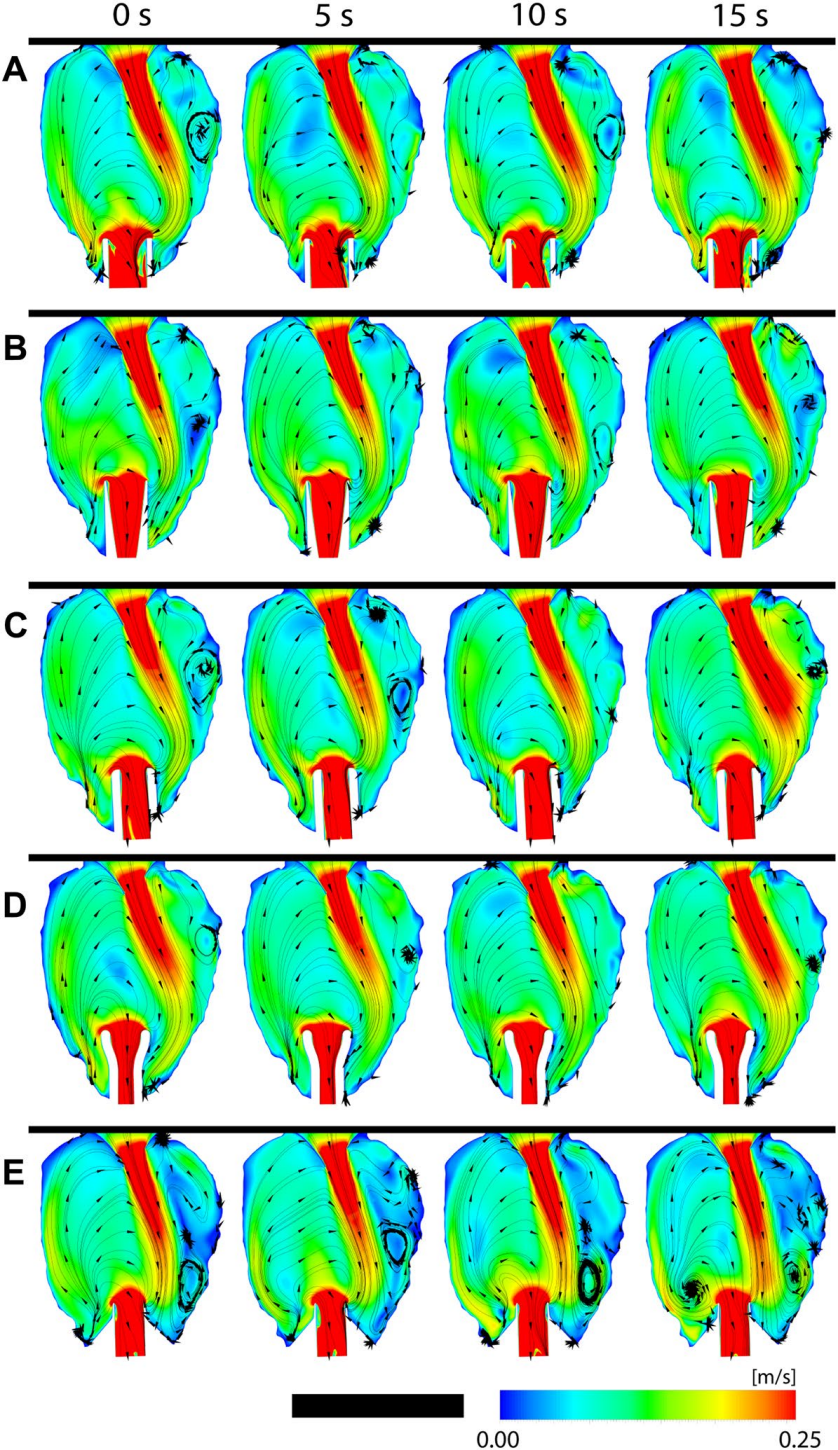
Velocity magnitude contour plot with velocity streamlines on the plane through the centre of all inflow cannulae. All cannulae resulted in a large clockwise fluid rotation under the anterior mitral valve leaflet. Cannula design references are labelled **A**–**E**, refer to Fig. 3. *Scale bar* represents 80 mm

Figure 5 shows a velocity magnitude contour plot with velocity streamlines on the plane through the centre of the IC. In all models, a MV jet can be seen to direct flow towards the apex. The maximum velocity of the MV jet in the anterior coronal plane shown in Fig. 5 was approximately 0.4 m/s. For the purpose of visualisation of the lower velocity scales, the maximum velocity in Fig. 5 was limited to 0.25 m/s. A large clockwise rotation was formed under the anterior MV leaflet with all models. Lower velocity flows were found under the posterior MV leaflet. Over the transient simulation, swaying of the mitral jet was observed in all cases. The average angle and standard deviation between the central axis of the IC and a line running through the origin of the mitral valve to the jet tip can be found in Table 2. It was found that the angles varied between 15.7 ° (cannula E) and 21 ° (cannula D). It appeared that cannula A, the trumpet tip, had the most stable jet and conversely, cannula C had the greatest variation. Even though a constant flow was prescribed, a transient behaviour was found. This could be due to the unstable self-interacting blood dynamics between the incoming jet and the pre-existing blood motion.

**Table 2:** Mitral jet movement

Model	Mean angle between jet and cannula (°)	Standard deviation (°)
A	20.2	0.6
B	19.5	1.7
C	19.3	2.5
D	21.0	0.9
E	15.7	1

**Fig. 6:**
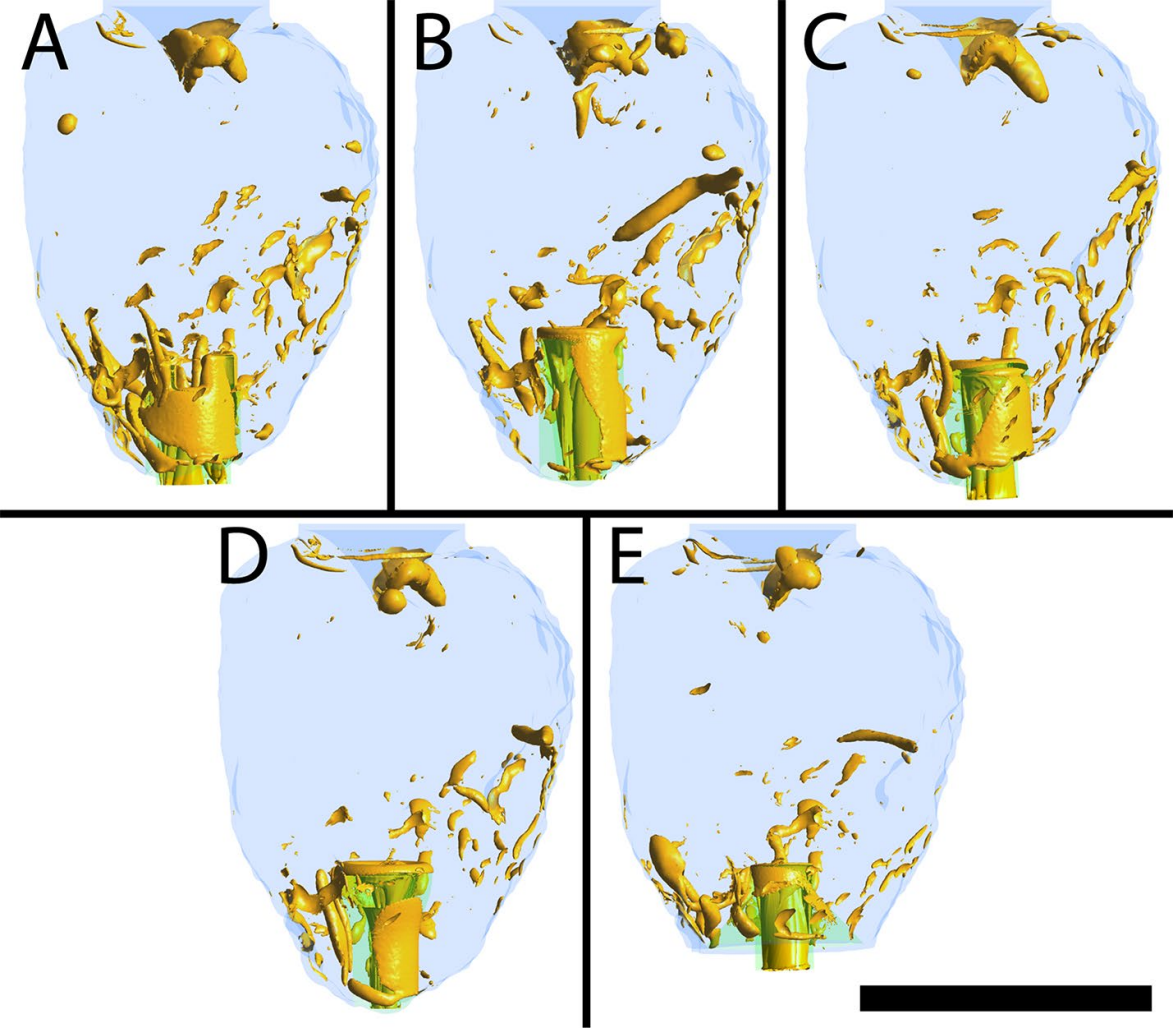
Vortex visualisation using the Q-criterion. All cannulae created unstructured vortices. Cannula design references are labelled **A**–**E**, refer to Fig. 3. *Scale bar* represents 70 mm

Vortices were determined using the Q-criterion [26]. The threshold for visualisation of vortex cores was defined at a value of 500 s^−2^, as seen in Fig. 6. In all cases, there were no apparent structured vortices or patterns within the LV.

### Stagnation regions

**Fig. 7:**
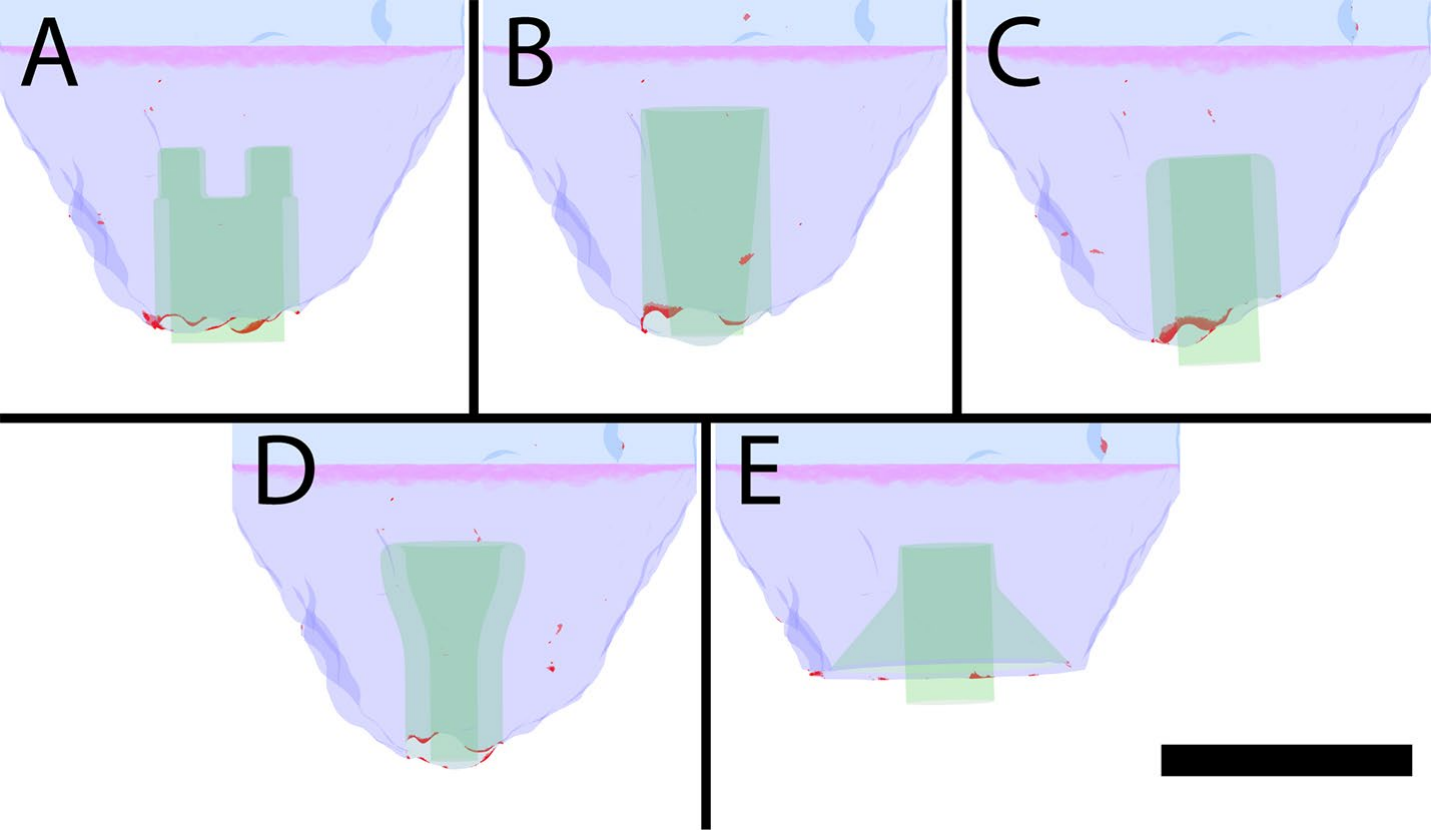
Thrombus risk rendering of the cannulation region with the persistent stagnation volumes shown in *red*. Thrombus risk was defined as velocity magnitudes of less than 0.001 m/s and strain rate of under 2/s. Cannula design references are labelled **A**–**E**, refer to Fig. 3. *Scale bar* represents 40 mm

Using the previously defined TR criteria, regions of low velocity and strain rate are highlighted in red which indicates areas of persistent stagnation and can be a source of thrombus formation, as illustrated in Fig. 7. Out of the five geometries studied, cannula C had the highest volumes of stagnant flow. In general, TR regions were seen to occur at the interface between the cannula and endocardium. Qualitatively comparing the cannulae in Fig. 7, it was found that cannula E had the lowest amount of persistent stagnation areas, which is possibly due to the smoother transition between the cannula and endocardium.

**Fig. 8:**
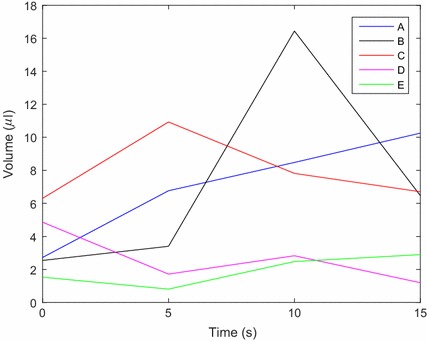
Total volume of thrombus risk regions over time for all cannulae within the defined volume of interest (*pink*), as shown in Fig. 7

Quantitatively, the volume of TR was calculated (Fig. 8) in the volume of interest highlighted in pink in Fig. 7. Confirming the qualitative findings, it was found that cannula E had the lowest average potential volume for thrombus formation at 2 µl. Conversely, cannula C had the highest risk of thrombus formation with a potential average volume of 8 µl. Cannulae a, b and d had an average TR volume of 7, 7 and 3 µl, respectively. The appearance and disappearance of TR volumes is illustrated in Fig. 8 as changes in TR volume. These low TR volumes can be appreciable as a study by Martin et al. [27] showed that thrombus emboli can be as small as 0.3 mm in diameter. Assuming a spherical emboli and a diameter of 0.3 mm, the volume of the thrombus emboli is approximately 0.014 µl.

### Volume of fluid washout model

**Fig. 9:**
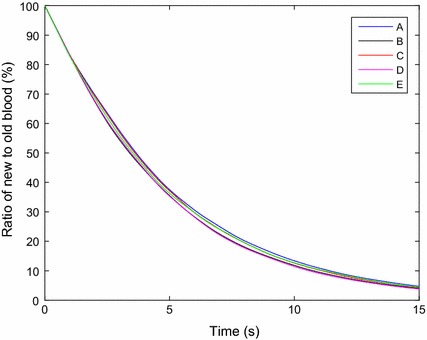
Comparison of the rate of wash out in the left ventricle using the volume of fluid model. The ratio is defined by the volume of old blood to the total blood volume of the respective model. At time 0 s, all models are filled with old blood and is replaced by new blood over time

**Fig. 10:**
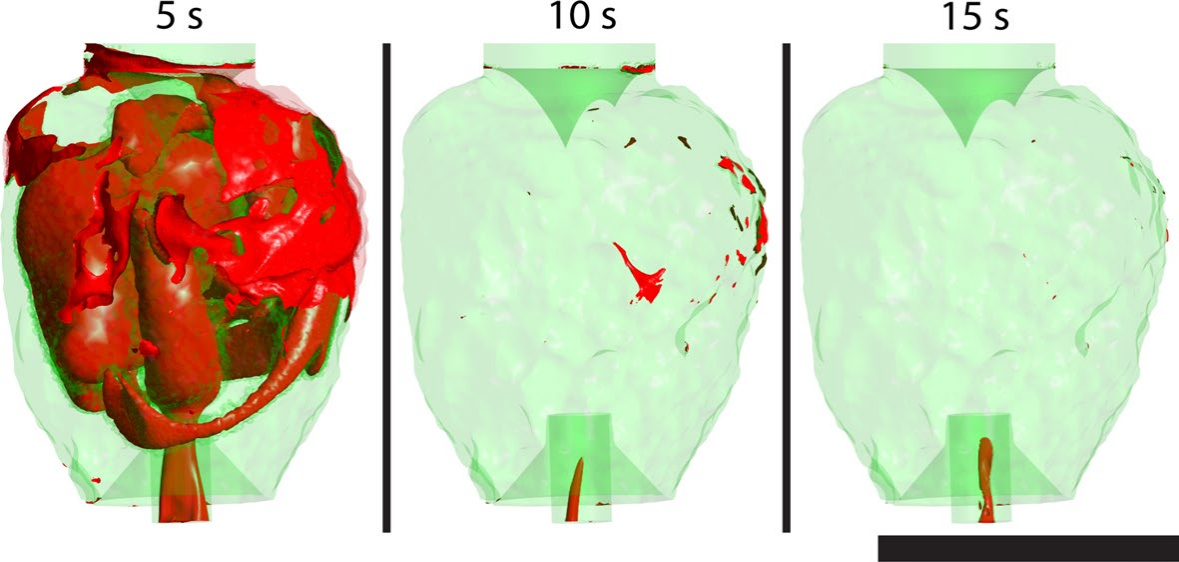
An example visualisation (cannula E) using the volume of fluid model showing old blood (*translucent red*) being replaced with new blood (*translucent green*). Interface between the old and new blood is shown as a *solid red* surface. Time 0 is not shown due the entire domain being filled with old blood. *Scale bar* represents 70 mm

The VOF washout model indicated that different cannula geometry does not have a significant influence on the rate of washout inside the LV. All cannulae were able to replace approximately 96% of the old blood in 15 s, as illustrated in Fig. 9. For visualisation purposes, the progression of old blood to new blood can be seen in Fig. 10 with cannula E. The old blood (volume fraction >0.5) has been coloured as translucent red with new blood (volume fraction <0.5) visualised as translucent green. The interface (volume fraction = 0.5) between the two phases is indicated with a red isosurface.

### Haemolysis index and blood residence time

**Fig. 11:**
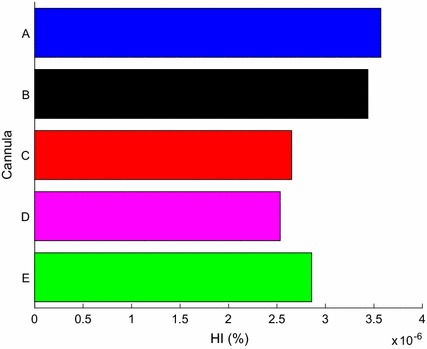
Haemolysis index sampled using mass-weighted average mid LV (50 mm above apex)

Evaluating the mass-weighted average HI on a transverse plane through the middle of the LV, it was found that cannula A had comparatively the highest HI (3.6e−6%), as seen in Fig. 11. The lowest HI (2.5e−6%) was found with cannula D. The two highest HI from cannulae A and B could be attributed to either the complex disturbances caused by the crown shaped tip and longer insertion lengths along with the sharp inflow inlet, respectively. Even though cannula D had a similar insertion length to cannula B, cannula d had the lowest HI and could be explained by the large curvature angles of cannula D.

**Fig. 12:**
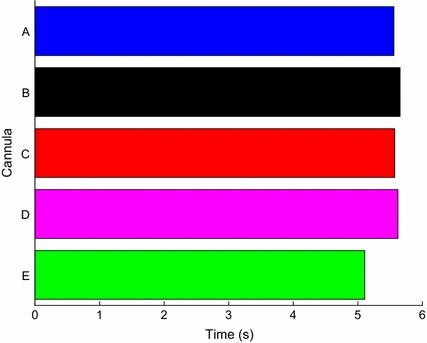
Blood residence time calculated using an Eulerian approach and sampled using an area-weighted average at the outlet

The average blood residence time through the LV using different cannulae varied between 5.1 and 5.7 s. The longest blood residence time was found when cannula B was used while cannula E had the shortest blood residence time. However, as seen in Fig. 12, all blood residence times were quite similar to each other and due to the transient flow, the average blood residence time was found not to be realistically different between the cannulae. As there was no pulsatility, along with the severely dilated LV, the blood residence time would be expected to be longer than healthy patients.

### Cannula wall shear stress

In all cases, the area-weighted average WSS oscillated over time. By taking the time averaged WSS, all cannulae were found to be significantly (p < 0.01, one-way ANOVA, Dunnett’s T3, IBM SPSS 23, New York, USA) different from each other. As shown in Table 3, four cannulae (A, C, D, E) were grouped in an average WSS range between 2.12 and 1.91 Pa. In this group, there was a maximum difference of 10%. However, cannula B was found to have a larger difference, a maximum of 47%, from the group of four cannulae that had larger inlet surface areas.

**Table 3:** Average wall shear stress on cannula surface

Model	Mean WSS (Pa)	Standard deviation (Pa)
A	2.12	0.08
B	1.11	0.08
C	2.08	0.04
D	1.97	0.07
E	1.91	0.15

## Discussion

This study aimed to determine the effects of different IC geometries on left intraventricular flow in terms of TR, BRT and LV washout. TR was defined by low velocity magnitudes coupled with low strain rate. In this study, it was shown that the inferiorly flared cannula has the potential to reduce incidences of thrombus formation with comparatively lower stagnation regions while all cannulae resulted in similar LV washout rates and blood residence times.

The implementation of a static approximation of a MV, developed by Domenichini et al. [19], was able to recreate general physiological flows. Intraventricular flow profiles were similar to a study by Bermejo et al. [28], where echocardiographic data was obtained in nonischemic dilated cardiomyopathy patients. A large dominant clockwise rotation was seen with all cannula cases, which agreed with the echocardiographic data during the diastasis phase [28]. Moreover, the low velocity region under the posterior MV leaflet agreed with the study by Bermejo et al. [28]. When using phase contrast magnetic imaging, it has been shown that a counter clockwise rotation forms during diastole near the apex [28]. Since the apex was cannulated in this study, a similar counter clockwise rotation of blood was seen superior to the LV apex and closer to the tip of the MV jet.

It has been suggested that the mid-diastolic vortices enhance blood mixing which can prevent stagnation areas [29]. Qualitatively evaluating the streamlines in Fig. 5, it can be seen that for cannula E, there were more distinct vortices near the ventricular apex which support the suggestion of enhanced blood mixing to reduce stagnation areas. Since this study modelled total heart failure, there were no distinct phases of the cardiac cycle. In the present work, all cannulae resulted in scattered vortex cores, which agrees with previous studies that have reported disturbed vortex structures in dilated ventricles [30–33].

The highest and most consistent areas of TR volume occurred at the interface between the cannula and endocardium where wedge thrombus has been reported in clinic [34]. Thrombus formation can be caused by hyper coagulability, stasis and endothelial injury, as described by Virchow’s triad [35]. As endothelial injury is already sustained around the ventricular apex, there is a need to reduce stasis or coagulability. Cannula E had the most potential to significantly reduce incidences of wedge thrombus formation by minimising areas of stasis, while its flared design may also cover the area of endothelial injury.

When comparing cannulae A, B and C to cannula E; the shorter cannula appeared to reduce stagnation areas as similarly reported by Ong et al. [13] and Antaki et al. [36]. However, cannula D had a similar length to cannula B and was found to have relatively low stagnation regions. This may indicate that cannula length is not the only dependent variable on stagnation regions: cannula geometry can have an effect.

The use of volume of fluid model was an efficient way to calculate LV washout. It was found that all cannulae had similar LV washout rates, indicating that different IC geometries do not significantly affect LV washout. It was expected that cannulae with higher stagnation regions would result in slower LV washout, however this was not found. This could be attributed to the low stagnation volumes and hence the volume of fluid may not have the sensitivity to detect such low volume differences. Furthermore, the blood residence time, using the Eulerian method, was found to be similar between the five cannulae, which supports the findings with similar rates of LV washout.

The highest time-averaged WSS was found with cannula A at 2.1 Pa, which is approximately 100 orders of magnitude lower than the suspected haemolysis threshold found by Reinhard et al. [37]. Between the highest (cannula A) and lowest (cannula B) time-averaged WSS, the difference was approximately 47%. The major difference between cannula A and B was that cannula A has a large number of surface features which are facing superiorly, representing a crown shape, while cannula B has a relatively sharp and simple inflow edge.

In general, the difference in HI due to different IC tips indicated that the complex crown shape (cannula A) and sharp inlet (cannula B) promotes relatively higher shear stresses within the LV as it has been found that the blood residence time was similar across all cannulae. It must be noted that the implemented HI method has been shown to have good correlation with experimental data but has large errors [24]. As a consequence, the HI prediction was used to relatively compare the models.

In summary, all cannulae had similar rates of LV washout and supported by similar blood residence times. The risk of thrombus formation due to blood stagnation is not solely dependent on cannula insertion length but also on cannula geometry. Cannula E was found to have the lowest risk of thrombus formation which can be attributed to the smoother transition from endocardium to the IC surface. As a result, it has been shown that the inferiorly flared cannula E has the potential to reduce the likelihood for wedge thrombus formation.

## Limitations

One major limitation of the presented model was the static LV and constant inflow. In most instances, some contractility of the LV is still present. The addition of a deforming wall boundary would change regions of possible stagnation and rate of LV washout. Meanwhile, implantation of LVADs are often performed with a functioning right ventricle. As a result, the incoming flow from the pulmonary vein would still have some pulsatility. This acceleration and deceleration of blood may affect the TR regions, LV washout and WSS on the cannula. Furthermore, the outlet boundary condition would not be 0 Pa in a pulsatile system, and a method to better represent physiological boundaries would be the coupling of a lumped parameter model, which numerically describes the cardiovascular system [38]. Clinically, the LVAD is often set to a speed where some aortic valve opening occurs to prevent valve fusion, aortic incompetence and reduction in thrombus potential [39]. As a result, an additional outlet boundary at the aorta could affect the intraventricular flow. Furthermore, a static MV was implemented. Due to adaptive ventricular remodelling from cardiac insufficiency, the geometry of the MV will change [40–42]. These changes could alter the intraventricular flow. The inclusion of realistic heart wall and valve movements is highly complex but can be achieved through fluid structure interactions or a prescribed moving mesh. The main limitation of such models is the excessive computational time required. Using such models to compare a large variety of cannulae would be computationally unfeasible at this time. This study did not take into account different apical thicknesses, which would affect insertion length. Future evaluations should consider insertion length and position, not just geometry. The presented CFD model did not include internal LV features but has been shown to increase the size of recirculation zones and deeper MV jet penetration [43].

Clinical translation of these results must first consider the given limitations. In the case of total heart failure with a severely dilated LV, the results from this study could provide a general overview of the intraventricular flow. This study can aid in the future design of inflow cannulae. It was found to be beneficial to minimise the inlet tip complexity, which can improve haemolysis indices within the LV. To minimise the risk of thrombus due to stagnation, an increase in angle between the endocardium and inflow cannula wall could be advantageous. Further validation of these recommendations will be required by using other techniques such as particle image velocimetry and in vivo validation.

## Conclusions

Five different IC were evaluated in a patient specific LV to determine blood stagnation regions, LV washout, relative blood damage indices, BRT, and average cannula WSS. General intraventricular flow features were able to be reproduced with an approximated MV. Areas of blood stagnation (TR volumes) were consistently found at the interface between the IC and endocardium. All cannulae had similar rates of LV washout and RBT. The complex crown tip results in relatively higher blood damage indices. However, all cannula WSS were below the haemolysis threshold. The use of an inferiorly flared IC has the potential to lower the incidences of thrombus formation.
